# Supplementary material for: Optimal dose of perineural dexamethasone for the prolongation of analgesia for peripheral nerve blocks: protocol for a systematic review and meta-analysis
Source: BMJ Open. 2023 Jul 19;13(7):e072598. doi: 10.1136/bmjopen-2023-072598 (PMC10357643; doi:10.1136/bmjopen-2023-072598)
Supplement: Supplementary data [file bmjopen-2023-072598supp001.pdf]

Search strategies

EMBASE

#1 ‘Dexamethasone’/exp OR ‘Dexamethasone’:ti,ab,kw

#2 ‘Nerve block’/exp OR ‘Nerve block’:ti,ab,kw OR ‘Nerve blockade’:ti,ab,kw OR ‘Peripheral nerve blockade’:ti,ab,kw OR ‘Peripheral nerve block’:ti,ab,kw

#3 ‘Randomized controlled trial’/exp OR ‘Randomized controlled trial’:ti,ab,kw OR ‘Clinical study’:ti,ab,kw OR ‘Controlled clinical trial’:ti,ab,kw OR ‘Clinical trial’:ti,ab,kw OR ‘Randomized’:ti,ab,kw

#4 #1 AND #2 AND #3

Cochrane library Trials

#1 MeSH descriptor: [Dexamethasone| explode all trees

#2 (Dexamethasone):ti,ab,kw

#3 #1 OR#2

#4 (Nerve block):ti,ab,kw

#5 (Nerve blockade):ti,ab,kw

#6 (Peripheral nerve blockade):ti,ab,kw

#7 (Peripheral nerve block):ti,ab,kw

#8 #4 OR #5 OR #6 OR #7

#9 (Randomized controlled trial):ti,ab,kw

#10 (Clinical study):ti,ab,kw

#11 (Controlled clinical trial):ti,ab,kw

#12 (Clinical trial):ti,ab,kw

#13 (Randomized):ti,ab,kw

#14 #9 OR #10 OR #11 OR #12 OR #13

#15 #3 AND #8 AND #14

Web of science

#1 Dexamethasone (Topic)

#2 Nerve block (Topic) OR Nerve blockade (Topic) OR Peripheral nerve blockade

(Topic) OR Peripheral nerve block (Topic)

#3 Randomized controlled trial (Topic) OR Clinical study (Topic) OR Controlled clinical trial (Topic) OR Clinical trial (Topic) OR Randomized (Topic)

#4 #1 AND #2 AND #3
