# Supplementary material for: Optimal dose of perineural dexamethasone for the prolongation of analgesia for peripheral nerve blocks: protocol for a systematic review and meta-analysis
Source: BMJ Open. 2023 Jul 19;13(7):e072598. doi: 10.1136/bmjopen-2023-072598 (PMC10357643; doi:10.1136/bmjopen-2023-072598)
Supplement: Author's manuscript [file bmjopen-2023-072598.draft_revisions.pdf]

BMJ Open is committed to open peer review. As part of this commitment we make the peer review history of every article we publish publicly available.

When an article is published we post the peer reviewers' comments and the authors' responses online. We also post the versions of the paper that were used during peer review. These are the versions that the peer review comments apply to.

The versions of the paper that follow are the versions that were submitted during the peer review process. They are not the versions of record or the final published versions. They should not be cited or distributed as the published version of this manuscript.

BMJ Open is an open access journal and the full, final, typeset and author-corrected version of record of the manuscript is available on our site with no access controls, subscription charges or pay-per-view fees (http://bmjopen.bmj.com).

If you have any questions on BMJ Open's open peer review process please email info.bmjopen@bmj.com

BMJ Open

Optimal dose of perineural dexamethasone regarding the efficacy and safety in peripheral nerve blocks: A protocol for systematic review and meta-analysis

Journal:	*BMJ Open*
Manuscript ID	bmjopen-2023-072598
Article Type:	Protocol
Date Submitted by the Author:	10-Feb-2023
Complete List of Authors:	Chai, Wenjie; Lanzhou Traditional Chinese Medicine Hospital of Orthopedics and Traumatology, Anesthesiology<br>Wang, Shaopeng; Lanzhou Traditional Chinese Medicine Hospital of Orthopedics and Traumatology, Anesthesiology<br>Zhang, Donghang; Sichuan University West China Hospital, Anesthesiology
Keywords:	Adult anaesthesia < ANAESTHETICS, Pain management < ANAESTHETICS, CLINICAL PHARMACOLOGY

SCHOLARONE™
Manuscripts

Optimal dose of perineural dexamethasone regarding the efficacy and safety in peripheral nerve blocks: A protocol for systematic review and meta-analysis

Wenjie Chai [1,*], Shaopeng Wang [1], Donghang Zhang [2,*]

[1] Department of Anesthesiology, Lanzhou Traditional Chinese Medicine Hospital of Orthopedics and Traumatology, Lanzhou, 730000, China.

[2] Department of Anesthesiology, West China Hospital of Sichuan University, Chengdu, 610041, China.

[*]**Address corresponding to:**

Dr. Wenjie Chai,

E-mail: 119251554@qq.com

Dr. Donghang Zhang

E-mail: zhangdhscu@163.com

For peer review only

For peer review only - http://bmjopen.bmj.com/site/about/guidelines.xhtml

Abstract

Introduction: Perineural use of dexamethasone is demonstrated to extend the analgesia duration of peripheral nerve blocks (PNB), but the optimal dose remains unclear. This protocol of a systematic review and meta-analysis aims to determine the optimal dose of perineural dexamethasone in the prolongation of analgesia for PNB.

Methods and analysis: PubMed, EMBASE, the Cochrane Central register of Controlled Trials (CENTRAL), and Web of science will be searched for randomized controlled trials (RCTs) that compared the efficacy and safety of different doses of perineural dexamethasone for PNB. The duration of analgesia will be defined as the primary outcome. Secondary outcomes will include pain scores, the total analgesics requirement over 48 hours, and the incidence of adverse effects. RevMan 5.3 software will be used for data analysis.

Ethics and dissemination: No ethical approval is required. The results of this study will be submitted to peer-review journals.

PROSPERO registration number: CRD42022385672.

Keywords: analgesia; dexamethasone; meta-analysis; protocol; peripheral nerve block

For peer review only

For peer review only - http://bmjopen.bmj.com/site/about/guidelines.xhtml

Strengths and limitations of this study

- The results of this study will provide guidelines for the dose selection of perineural dexamethasone for PNB.
- The results of subgroup analysis may recommend the optimal dose of perineural dexamethasone for specific type of PNB.
- The results of this study may facilitate more well-designed RCTs to be conducted to confirm the optimal dose of perineural dexamethasone for PNB.
- High clinical heterogeneity may exist due to several factors, such us the type and concentration of local anesthetics, the type of surgery, anesthesia, and nerve blocks.

For peer review only

For peer review only - http://bmjopen.bmj.com/site/about/guidelines.xhtml

Introduction

Peripheral nerve block (PNB) is widely used for anesthesia and/or analgesia, however, the analgesic effects of single-shot PNB last only a few hours. [1] Several adjuvants have been used to prolong the analgesic duration of PNB, including intravenous or perineural injection of dexamethasone, dexmedetomidine, and opioids, [2 3] of which dexamethasone is the most commonly used adjuvant. [4 5] Perineural use of dexamethasone is used more commonly for PNB than that of intravenous route, and a previous meta-analysis suggests that perineural dexamethasone provides a longer duration of analgesia compared with intravenous dexamethasone when used as an adjuvant in PNB. [4 6] Currently, effects of various doses of perineural dexamethasone on PNB, such as 1 mg, 2 mg, 4 mg, 5 mg, and 8 mg have been investigated and compared, [7-20] the results remain controversial. Furthermore, previous studies have showed that perineural dexamethasone has a ceiling dose of 4 mg for prolongation of analgesia duration when used in combination with local anesthetics for PNB. [16] Therefore, it is worthwhile to conduct a systematic review and meta-analysis to determine the optimal dose of perineural dexamethasone in analgesic effects and safety for PNB.

For peer review only

For peer review only - http://bmjopen.bmj.com/site/about/guidelines.xhtml

Materials and analysis

Patient and Public Involvement

Patients and the public were not involved in the design, conduct, reporting, or dissemination plans of this study.

Study registration

This protocol has been registered in the International Prospective Register of Systematic Reviews (PROSPERO; registration number: CRD42022385672) and is conducted according to the Preferred Reporting Items for Systematic Evaluation and Meta-Analysis (PRISMA-P) Protocols.

Search strategy

Two authors will perform a comprehensive search in PubMed, EMBASE, CENTRAL, and Web of science from inception to March 1, 2023 for RCTs comparing the analgesic effects of various doses of perineural dexamethasone in PNB. Language will be restricted in English. The key terms used in our systematic search will include "Dexamethasone", "Peripheral nerve block", and "Randomized controlled trials". We will also track additional studies by reviewing the references of relative studies. The search strategy for PubMed is shown in Table 1.

Inclusion and exclusion

Inclusion criteria: (a) Study design: RCTs, (b) Participants: Adult patients (> 18 years) with PNB, (c) Comparisons: different doses of perineural dexamethasone as an adjuvant to local anesthetics, and (d) Primary outcomes: Duration of analgesia; Secondary outcomes: Pain scores, total analgesics requirement over 48 hours, and adverse effects (e.g., nausea, vomiting, drowsiness, dizziness and constipation). Retrospective studies, reviews, meta-analysis, case reports, conference abstracts, comments, and studies regarding pediatric surgeries will be excluded.

Study selection

Two authors will independently identify the eligible studies by screening the title, abstract and then reviewing the full-text of potentially eligible articles. Disagreements will be resolved by discussion with a third author.

Data extraction

For peer review only - http://bmjopen.bmj.com/site/about/guidelines.xhtml

Two authors will independently extract the following information from included studies: authors, publication year, countries, sample size, patients' age, types of surgery, types of anesthesia and nerve block, interventions, controls, outcomes, and postoperative analgesia. We will attempt to contact the corresponding author for raw data if the results were reported by median with range. If there is no response, the median with range will be converted to mean with standard deviation according to the methods described by Hozo et al [21]. Disagreements will be discussed with a third author.

Risk of bias assessment

Two authors will independently assess the quality of included studies using the Cochrane Collaboration's tool for risk of bias [22]. The assessment for risk of bias contains six items: (a) random sequence generation (selection bias); (b) allocation concealment (selection bias); (c) blinding of participants and personnel (performance bias); (d) blinding of outcome assessment (detection bias); (e) incomplete outcome data (attrition bias); and (f) selective reporting (reporting bias). The assessment for each item will be described as 'low', 'high', or 'unclear'. Disagreements will be discussed with a third author.

Statistical analysis

The Mean differences (MD) and risk ratios (RR) with 95% confidence intervals (CI) will be calculated for continuous and dichotomous data, respectively. We will use random-effect model to synthesize data due to the clinical heterogeneity (e.g., type of surgery, anesthesia, and peripheral nerve block). Statistical heterogeneity will be assessed by the I^2 test. Significant heterogeneity will be considered existed when I^2 statistic > 50%. Subgroup analysis will be performed to explore the source of heterogeneity among studies. $P < 0.05$ is considered statistically significant. The quality of evidence will be rated as 'high', 'moderate', 'low', or 'very low' using the GRADE approach.

For peer review only - http://bmjopen.bmj.com/site/about/guidelines.xhtml

Discussion

Currently, various doses of perineural dexamethasone have been used in PNB to extend the duration of analgesia. Considering the potential neurotoxicity for perineural use of high dose of dexamethasone, it is meaningful to explore the optimal dose of perineural dexamethasone which will provide longer analgesia and lower incidence of adverse effects. This present protocol of a systematic review and meta-analysis will investigate whether there is a dose-dependency and explore the optimal dose of perineural dexamethasone for PNB. Therefore, the results of our study may provide guidelines for dose selection of perineural dexamethasone for PNB. Moreover, from the results of subgroup analysis, we may recommend the optimal dose of perineural dexamethasone for specific type of PNB. Additionally, our results may facilitate more well-designed RCTs to be conducted to confirm the optimal dose of perineural dexamethasone for PNB.

For peer review only

For peer review only - http://bmjopen.bmj.com/site/about/guidelines.xhtml

Ethics and dissemination

No ethical approval is required for this study. The results of this study will be submitted to peer-review journals.

Author Contributions

Conceptualization: Wenjie Chai and Donghang Zhang.

Data curation: Shaopeng Wang.

Formal analysis: Wenjie Chai.

Methodology: Wenjie Chai, Shaopeng Wang.

Validation: Wenjie Chai, Shaopeng Wang.

Writing – original draft: Wenjie Chai and Donghang Zhang.

Writing – review & editing: Wenjie Chai.

Competing interests

None.

Funding

This study was supported by Grant No. 2022NSFSC1399 (To Donghang Zhang) from the Natural Science Foundation of Sichuan Province.

Acknowledgments

None.

For peer review only - http://bmjopen.bmj.com/site/about/guidelines.xhtml

References

1. Pehora C, Pearson AM, Kaushal A, et al. Dexamethasone as an adjuvant to peripheral nerve block. *Cochrane Database Syst Rev* 2017;11(11):Cd011770. doi: 10.1002/14651858.CD011770.pub2 [published Online First: 2017/11/10]

2. Desai N, Kirkham KR, Albrecht E. Local anaesthetic adjuncts for peripheral regional anaesthesia: a narrative review. *Anaesthesia* 2021;76 Suppl 1:100-09. doi: 10.1111/anae.15245 [published Online First: 2021/01/12]

3. Xuan C, Yan W, Wang D, et al. The Facilitatory Effects of Adjuvant Pharmaceutics to Prolong the Duration of Local Anesthetic for Peripheral Nerve Block: A Systematic Review and Network Meta-analysis. *Anesth Analg* 2021 doi: 10.1213/ane.0000000000005640 [published Online First: 2021/06/22]

4. Zorrilla-Vaca A, Li J. Dexamethasone Injected Perineurally is More Effective than Administered Intravenously for Peripheral Nerve Blocks: A Meta-Analysis of Randomized Controlled Trials. *Clin J Pain* 2018;34(3):276-84. doi: 10.1097/ajp.0000000000000519 [published Online First: 2017/06/08]

5. Zhang D, Zhou C, Wei D, et al. Dexamethasone added to local anesthetics in ultrasound-guided transversus abdominis plain (TAP) block for analgesia after abdominal surgery: A systematic review and meta-analysis of randomized controlled trials. *PLoS One* 2019;14(1):e0209646. doi: 10.1371/journal.pone.0209646 [published Online First: 2019/01/09]

6. Heesen M, Klimek M, Imberger G, et al. Co-administration of dexamethasone with peripheral nerve block: intravenous vs perineural application: systematic review, meta-analysis, meta-regression and trial-sequential analysis. *Br J Anaesth* 2018;120(2):212-27. doi: 10.1016/j.bja.2017.11.062 [published Online First: 2018/02/07]

7. Short A, El-Boghdadly K, Clarke H, et al. Effect of intravenous dexamethasone on the anaesthetic characteristics of peripheral nerve block: a double-blind, randomised controlled, dose-response volunteer study. *Br J Anaesth* 2020;124(1):92-100. doi: 10.1016/j.bja.2019.08.029 [published Online First: 2019/11/13]

For peer review only - http://bmjopen.bmj.com/site/about/guidelines.xhtml

8. McHardy PG, Singer O, Awad IT, et al. Comparison of the effects of perineural or intravenous dexamethasone on low volume interscalene brachial plexus block: a randomised equivalence trial. *Br J Anaesth* 2020;124(1):84-91. doi: 10.1016/j.bja.2019.08.025 [published Online First: 2019/10/09]

9. Fang J, Shi Y, Du F, et al. The effect of perineural dexamethasone on rebound pain after ropivacaine single-injection nerve block: a randomized controlled trial. *BMC Anesthesiol* 2021;21(1):47. doi: 10.1186/s12871-021-01267-z [published Online First: 2021/02/14]

10. Ibrahim AS, Aly MG, Farrag WS, et al. Ultrasound-guided adductor canal block after arthroscopic anterior cruciate ligament reconstruction: Effect of adding dexamethasone to bupivacaine, a randomized controlled trial. *Eur J Pain* 2019;23(1):135-41. doi: 10.1002/ejp.1292 [published Online First: 2018/08/02]

11. Abdel-wahab AH, Osman EA, Ahmed AY. Comparison of postoperative analgesic effects of two doses of dexamethasone in ultrasound-guided transversus abdominis plane block for inguinal hernia repair: a randomized controlled trial. *Ain Shams Journal of Anesthesiology* 2021;13(1) doi: 10.1186/s42077-021-00139-5

12. Acharya R, Sriramka B, Panigrahi S. Comparison of 4 mg dexamethasone versus 8 mg dexamethasone as an adjuvant to levobupivacaine in fascia iliaca block - A prospective study. *The korean journal of pain* 2018;31(4):261-67. doi: 10.3344/kjp.2018.31.4.261

13. Holland D, Amadeo RJJ, Wolfe S, et al. Effect of dexamethasone dose and route on the duration of interscalene brachial plexus block for outpatient arthroscopic shoulder surgery: a randomized controlled trial. *Canadian Journal of Anesthesia-Journal Canadien D Anesthesie* 2018;65(1):34-45. doi: 10.1007/s12630-017-0989-7

14. Tandoc MN, Fan L, Kolesnikov S, et al. Adjuvant dexamethasone with bupivacaine prolongs the duration of interscalene block: a prospective randomized trial. *Journal of anesthesia* 2011;25(5):704-09. doi: 10.1007/s00540-011-1180-x

15. Gendy H, Sharnouby NE. Ultrasound-guided single injection transversus abdominis

For peer review only - http://bmjopen.bmj.com/site/about/guidelines.xhtml

plane block of isobaric bupivacaine with or without dexamethasone for bariatric patients undergoing laparoscopic vertical banded gastroplasty: a comparative study of different doses. *Ain-Shams Journal of Anaesthesiology* 2015;8(2):194-.

16. Albrecht E, Reynvoet M, Fournier N, et al. Dose-response relationship of perineural dexamethasone for interscalene brachial plexus block: a randomised, controlled, triple-blind trial. *Anaesthesia* 2019;74(8):1001-08. doi: 10.1111/anae.14650 [published Online First: 2019/04/12]

17. Bravo D, Aliste J, Layera S, et al. A multicenter, randomized comparison between 2, 5, and 8 mg of perineural dexamethasone for ultrasound-guided infraclavicular block. *Reg Anesth Pain Med* 2019;44(1):46-51. doi: 10.1136/rapm-2018-000032 [published Online First: 2019/01/15]

18. Liu J, Richman KA, Grodofsky SR, et al. Is there a dose response of dexamethasone as adjuvant for supraclavicular brachial plexus nerve block? A prospective randomized double-blinded clinical study. *J Clin Anesth* 2015;27(3):237-42. doi: 10.1016/j.jclinane.2014.12.004 [published Online First: 2015/02/02]

19. Turner JD, Henshaw DS, Weller RS, et al. Perineural dexamethasone successfully prolongs adductor canal block when assessed by objective pinprick sensory testing: A prospective, randomized, dose-dependent, placebo-controlled equivalency trial. *J Clin Anesth* 2018;48:51-57. doi: 10.1016/j.jclinane.2018.05.009 [published Online First: 2018/05/13]

20. Woo JH, Kim YJ, Kim DY, et al. Dose-dependency of dexamethasone on the analgesic effect of interscalene block for arthroscopic shoulder surgery using ropivacaine 0.5%: A randomised controlled trial. *Eur J Anaesthesiol* 2015;32(9):650-5. doi: 10.1097/eja.0000000000000213 [published Online First: 2015/01/21]

21. Hozo SP, Djulbegovic B, Hozo I. Estimating the mean and variance from the median, range, and the size of a sample. *BMC Med Res Methodol* 2005;5:13. doi: 10.1186/1471-2288-5-13 [published Online First: 2005/04/21]

22. Higgins J, Green SR. Cochrane Handbook for Systematic Review of InterventionsVersion 5.1.0. 2011

For peer review only

1

2

3

For peer review only

For peer review only - http://bmjopen.bmj.com/site/about/guidelines.xhtml

Table 1. Search strategy for PubMed.

Number	Search terms
#1	Dexamethasone [Mesh]
#2	Dexamethasone [Title/Abstract]
#3	#1 OR #2
#4	Nerve block [Title/Abstract]
#5	Nerve blockade [Title/Abstract]
#6	Peripheral nerve blockade [Title/Abstract]
#7	Peripheral nerve block [Title/Abstract]
#8	#4 OR #5 OR #6 OR #7
#9	Randomized controlled trial [Title/Abstract]
#10	Clinical study [Title/Abstract]
#11	Controlled clinical trial [Title/Abstract]
#12	Clinical trial [Title/Abstract]
#13	Randomized [Title/Abstract]
#14	#9 OR #10 OR #11 OR #12 OR #13
#15	#3 AND #8 AND #14

For peer review only

For peer review only - http://bmjopen.bmj.com/site/about/guidelines.xhtml

BMJ Open

Optimal dose of perineural dexamethasone for the prolongation of analgesia for peripheral nerve blocks: protocol for a systematic review and meta-analysis

Journal:	*BMJ Open*
Manuscript ID	bmjopen-2023-072598.R1
Article Type:	Protocol
Date Submitted by the Author:	09-Jun-2023
Complete List of Authors:	Chai, Wenjie; Lanzhou Traditional Chinese Medicine Hospital of Orthopedics and Traumatology, Anesthesiology<br>Wang, Shaopeng; Lanzhou Traditional Chinese Medicine Hospital of Orthopedics and Traumatology, Anesthesiology<br>Zhang, Donghang; Sichuan University West China Hospital, Anesthesiology
**Primary Subject Heading**:	Anaesthesia
Secondary Subject Heading:	Anaesthesia
Keywords:	Adult anaesthesia < ANAESTHETICS, Pain management < ANAESTHETICS, CLINICAL PHARMACOLOGY

SCHOLARONE™
Manuscripts

Optimal dose of perineural dexamethasone for the prolongation of analgesia for peripheral nerve blocks: protocol for a systematic review and meta-analysis

Wenjie Chai [1, *], Shaopeng Wang [1], Donghang Zhang [2, *]

[1] Department of Anesthesiology, Lanzhou Traditional Chinese Medicine Hospital of Orthopedics and Traumatology, Lanzhou, 730000, China.

[2] Department of Anesthesiology, West China Hospital of Sichuan University, Chengdu, 610041, China.

***Correspondence to:**

Dr. Wenjie Chai

E-mail: 119251554@qq.com

Dr. Donghang Zhang

E-mail: zhangdhscu@163.com

For peer review only - http://bmjopen.bmj.com/site/about/guidelines.xhtml

Abstract

Introduction: Perineural use of dexamethasone is demonstrated to extend the analgesia duration of peripheral nerve blocks (PNB), but its optimal dose remains unclear. This systematic review and meta-analysis aims to determine the optimal dose of perineural dexamethasone in the prolongation of analgesia for PNB.

Methods and analysis: PubMed, EMBASE, the Cochrane Central Register of Controlled Trials (CENTRAL), and Web of Science will be searched from their inception to March 1, 2023. Language will be restricted in English. Randomized controlled trials (RCTs) that compared the efficacy and safety of different doses of perineural dexamethasone for PNB in adult patients will be included. Retrospective studies, reviews, meta-analysis, case reports, conference abstracts, comments, and studies regarding pediatric surgeries will be excluded. The duration of analgesia will be defined as the primary outcome. Secondary outcomes will include pain scores, the total analgesics requirement over 48 hours, and the incidence of adverse effects. Two reviewers will independently perform the study selection, data extraction and quality assessment. RevMan 5.3 software will be used for data analysis. The quality of evidence will be assessed using the GRADE approach.

Ethics and dissemination: No ethical approval is required. The results of this study will be submitted to peer-review journals.

PROSPERO registration: CRD42022385672.

Keywords: analgesia; dexamethasone; meta-analysis; protocol; peripheral nerve block

For peer review only

Strengths and limitations of this study

- The GRADE approach will be used to assess the quality of evidence.
- Subgroup analysis, meta-regression, and sensitivity analyses will be performed to explore heterogeneity, and to attempt to identify the optimal dose of perineural dexamethasone for specific type of peripheral nerve block.
- High clinical heterogeneity may exist due to several factors, such as the type and concentration of local anesthetics, type of surgery, anesthesia, and nerve blocks.

For peer review only

For peer review only - http://bmjopen.bmj.com/site/about/guidelines.xhtml

Introduction

Peripheral nerve block (PNB) is widely used for anesthesia and/or analgesia, however, the analgesic effects of single-shot PNB last only a few hours. [1] Several adjuvants have been used to prolong the analgesic duration of PNB, including intravenous or perineural injection of dexamethasone, dexmedetomidine, and opioids. [2 3] Dexamethasone is the most commonly used adjuvant for PNB. [4 5] Perineural use of dexamethasone is used more commonly for PNB than the intravenous route, and a previous meta-analysis suggested that perineural dexamethasone provided a longer duration of analgesia compared with intravenous dexamethasone when used as an adjuvant in PNB. [4 6] Currently, many studies have compared the effects of various doses of perineural dexamethasone on PNB, such as 1 mg, 2 mg, 4 mg, 5 mg, and 8 mg, [7-20] but the results remain controversial. Two previous meta-analysis [21 22] have evaluated the effects of different doses of perineural dexamethasone on PNB. One, published in 2015, showed no differences in the safety and efficacy between 4 and 8 mg perineural dexamethasone for PNB. [22] Another, published in 2018, reported that 4 mg of perineural dexamethasone represented a ceiling dose for the prolongation of analgesic duration when used in combination with local anesthetics for PNB. [21] Recently, several new RCTs that comparing the effects of different doses of perineural dexamethasone on PNB have been reported, but yielded inconsistent results. [12 13 16 23-25] Therefore, it is appropriate to conduct an updated systematic review and meta-analysis to provide the latest evidence about the optimal dose of perineural dexamethasone for PNB.

For peer review only - http://bmjopen.bmj.com/site/about/guidelines.xhtml

Methods and analysis

Study registration

This systematic review has been registered with the International Prospective Register of Systematic Reviews (PROSPERO; registration number: CRD42022385672) and is reported according to the Preferred Reporting Items for Systematic review and Meta-Analysis Protocols (PRISMA-P) guidance.

Search strategy

Two authors will perform a comprehensive search in PubMed, EMBASE, the Cochrane Central Register of Controlled Trials (CENTRAL), and Web of Science from inception to March 1, 2023 to identify RCTs comparing the analgesic effects of various doses of perineural dexamethasone in PNB. Language will be restricted in English. The key terms used in our systematic search will include "Dexamethasone", "Peripheral nerve block", and "Randomized controlled trials". We will also track additional studies by reviewing the references of relative studies. The search strategy for PubMed is shown in Table 1 and the search strategies for other databases are presented in Supplementary file 1.

Table 1. Search strategy for PubMed

	**Search terms**
#1	Dexamethasone [Mesh]
#2	Dexamethasone [Title/Abstract]
#3	#1 OR #2
#4	Nerve block [Title/Abstract]
#5	Nerve blockade [Title/Abstract]
#6	Peripheral nerve blockade [Title/Abstract]
#7	Peripheral nerve block [Title/Abstract]
#8	#4 OR #5 OR #6 OR #7
#9	Randomized controlled trial [Title/Abstract]
#10	Clinical study [Title/Abstract]

For peer review only - http://bmjopen.bmj.com/site/about/guidelines.xhtml

#11	Controlled clinical trial [Title/Abstract]
#12	Clinical trial [Title/Abstract]
#13	Randomized [Title/Abstract]
#14	#9 OR #10 OR #11 OR #12 OR #13
#15	#3 AND #8 AND #14

Inclusion and exclusion

Inclusion criteria: (a) Study design: RCTs, (b) Participants: Adult patients (> 18 years) with PNB, (c) Comparisons: Different doses of perineural dexamethasone as an adjuvant to local anesthetics, and (d) Primary outcome: Duration of analgesia; Secondary outcomes: Pain scores, total analgesics requirement over 48 hours, and adverse effects (e.g., neuropraxia, nausea, vomiting, drowsiness, dizziness and constipation). Retrospective studies, reviews, meta-analysis, case reports, conference abstracts, comments, and studies regarding pediatric surgeries will be excluded.

Study selection

Two authors will independently identify the eligible studies by screening the title and abstract, followed by reviewing the full texts of potentially eligible articles. Disagreements will be resolved by discussion with a third author. Study selection will be reported using a PRISMA flowchart (Figure 1).

Data extraction

Two authors will independently extract the following information from included studies: authors, publication year, countries, sample size, patients' age, Body Mass Index (BMI), the concentration of dexamethasone, types of surgery, types of anesthesia and nerve block, interventions, controls, outcomes, and postoperative analgesia. We will attempt to contact the corresponding author for raw data if the results were reported by median with range. If there is no response, the median with range will be converted to mean with standard deviation according to the methods described by Hozo et al. [26] Disagreements will be discussed with a third author.

Risk of bias assessment

For peer review only - http://bmjopen.bmj.com/site/about/guidelines.xhtml

Two authors will independently assess the quality of included studies using the Cochrane Collaboration's tool for risk of bias [27]. The assessment for risk of bias contains six items: (a) random sequence generation (selection bias); (b) allocation concealment (selection bias); (c) blinding of participants and personnel (performance bias); (d) blinding of outcome assessment (detection bias); (e) incomplete outcome data (attrition bias); and (f) selective reporting (reporting bias). The assessment for each item will be described as 'low', 'high', or 'unclear'. Disagreements will be discussed with a third author.

Statistical analysis

The Mean differences (MD) and risk ratios (RR) with 95% confidence intervals (CI) will be calculated for continuous and dichotomous data, respectively. We will use the random-effect model to synthesize data due to the clinical heterogeneity (e.g., type of surgery, anesthesia, and peripheral nerve block). Statistical heterogeneity will be assessed by the I^2 test. Significant heterogeneity will be considered existed when I^2 statistic > 50%. Subgroup analysis and meta-regression will be performed to explore the source of heterogeneity among studies. Sensitivity analyses will be conducted to assess whether the pooled results are robust. $P < 0.05$ is considered statistically significant. The quality of evidence will be rated as 'high', 'moderate', 'low', or 'very low' using the GRADE approach.

Patient and Public Involvement

None.

For peer review only - http://bmjopen.bmj.com/site/about/guidelines.xhtml

Ethics and dissemination

No ethical approval is required for this study. The results of this study will be submitted to peer-review journals.

Discussion

Currently, various doses of perineural dexamethasone have been used in PNB to extend the duration of analgesia. Considering the potential neurotoxicity for perineural use of high dose of dexamethasone, it is meaningful to explore the optimal dose of perineural dexamethasone for PNB which will provide longer analgesia and lower incidence of adverse effects. This present protocol of a systematic review and meta-analysis will investigate whether there is a dose-dependency for perineural dexamethasone on the analgesic effects and explore its optimal dose for PNB. Therefore, the results of our study may provide guidelines for dose selection of perineural dexamethasone for PNB. However, high clinical heterogeneity may exist due to several factors, such us the type and concentration of local anesthetics, the type of surgery, anesthesia, and nerve blocks. Therefore, we will perform subgroup analysis and meta-regression to explore the heterogeneity resource. Moreover, sensitivity analyses will also be conducted to test whether the pooled results are robust. From the results of subgroup analysis, we may recommend the optimal dose of perineural dexamethasone for specific local anesthetic in specific type of PNB. Additionally, our results may facilitate more well-designed RCTs to be conducted to confirm the optimal dose of perineural dexamethasone for PNB.

For peer review only - http://bmjopen.bmj.com/site/about/guidelines.xhtml

Contributors

Conceptualization: Wenjie Chai and Donghang Zhang. Data curation: Shaopeng Wang. Formal analysis: Wenjie Chai. Methodology: Wenjie Chai, Shaopeng Wang. Validation: Wenjie Chai, Shaopeng Wang. Writing (original draft): Wenjie Chai and Donghang Zhang. Writing (review & editing): Wenjie Chai.

Competing interests

None.

Funding

This study is supported by Grant No. 2022NSFSC1399 (To Donghang Zhang) from the Natural Science Foundation of Sichuan Province.

For peer review only

References

1. Pehora C, Pearson AM, Kaushal A, et al. Dexamethasone as an adjuvant to peripheral nerve block. *Cochrane Database Syst Rev* 2017;11(11):Cd011770. doi: 10.1002/14651858.CD011770.pub2 [published Online First: 2017/11/10]
2. Desai N, Kirkham KR, Albrecht E. Local anaesthetic adjuncts for peripheral regional anaesthesia: a narrative review. *Anaesthesia* 2021;76 Suppl 1:100-09. doi: 10.1111/anae.15245 [published Online First: 2021/01/12]
3. Xuan C, Yan W, Wang D, et al. The Facilitatory Effects of Adjuvant Pharmaceutics to Prolong the Duration of Local Anesthetic for Peripheral Nerve Block: A Systematic Review and Network Meta-analysis. *Anesth Analg* 2021 doi: 10.1213/ane.0000000000005640 [published Online First: 2021/06/22]
4. Zorrilla-Vaca A, Li J. Dexamethasone Injected Perineurally is More Effective than Administered Intravenously for Peripheral Nerve Blocks: A Meta-Analysis of Randomized Controlled Trials. *Clin J Pain* 2018;34(3):276-84. doi: 10.1097/ajp.0000000000000519 [published Online First: 2017/06/08]
5. Zhang D, Zhou C, Wei D, et al. Dexamethasone added to local anesthetics in ultrasound-guided transversus abdominis plain (TAP) block for analgesia after abdominal surgery: A systematic review and meta-analysis of randomized controlled trials. *PLoS One* 2019;14(1):e0209646. doi: 10.1371/journal.pone.0209646 [published Online First: 2019/01/09]
6. Heesen M, Klimek M, Imberger G, et al. Co-administration of dexamethasone with peripheral nerve block: intravenous vs perineural application: systematic review, meta-analysis, meta-regression and trial-sequential analysis. *Br J Anaesth* 2018;120(2):212-27. doi: 10.1016/j.bja.2017.11.062 [published Online First: 2018/02/07]
7. Short A, El-Boghdadly K, Clarke H, et al. Effect of intravenous dexamethasone on the anaesthetic characteristics of peripheral nerve block: a double-blind, randomised controlled, dose-response volunteer study. *Br J Anaesth* 2020;124(1):92-100. doi: 10.1016/j.bja.2019.08.029 [published Online First: 2019/11/13]

For peer review only - http://bmjopen.bmj.com/site/about/guidelines.xhtml

8. McHardy PG, Singer O, Awad IT, et al. Comparison of the effects of perineural or intravenous dexamethasone on low volume interscalene brachial plexus block: a randomised equivalence trial. *Br J Anaesth* 2020;124(1):84-91. doi: 10.1016/j.bja.2019.08.025 [published Online First: 2019/10/09]

9. Fang J, Shi Y, Du F, et al. The effect of perineural dexamethasone on rebound pain after ropivacaine single-injection nerve block: a randomized controlled trial. *BMC Anesthesiol* 2021;21(1):47. doi: 10.1186/s12871-021-01267-z [published Online First: 2021/02/14]

10. Ibrahim AS, Aly MG, Farrag WS, et al. Ultrasound-guided adductor canal block after arthroscopic anterior cruciate ligament reconstruction: Effect of adding dexamethasone to bupivacaine, a randomized controlled trial. *Eur J Pain* 2019;23(1):135-41. doi: 10.1002/ejp.1292 [published Online First: 2018/08/02]

11. Abdel-wahab AH, Osman EA, Ahmed AY. Comparison of postoperative analgesic effects of two doses of dexamethasone in ultrasound-guided transversus abdominis plane block for inguinal hernia repair: a randomized controlled trial. *Ain Shams Journal of Anesthesiology* 2021;13(1) doi: 10.1186/s42077-021-00139-5

12. Acharya R, Sriramka B, Panigrahi S. Comparison of 4 mg dexamethasone versus 8 mg dexamethasone as an adjuvant to levobupivacaine in fascia iliaca block - A prospective study. *The korean journal of pain* 2018;31(4):261-67. doi: 10.3344/kjp.2018.31.4.261

13. Holland D, Amadeo RJJ, Wolfe S, et al. Effect of dexamethasone dose and route on the duration of interscalene brachial plexus block for outpatient arthroscopic shoulder surgery: a randomized controlled trial. *Canadian Journal of Anesthesia-Journal Canadien D Anesthesie* 2018;65(1):34-45. doi: 10.1007/s12630-017-0989-7

14. Tandoc MN, Fan L, Kolesnikov S, et al. Adjuvant dexamethasone with bupivacaine prolongs the duration of interscalene block: a prospective randomized trial. *Journal of anesthesia* 2011;25(5):704-09. doi: 10.1007/s00540-011-1180-x

15. Gendy H, Sharnouby NE. Ultrasound-guided single injection transversus abdominis

For peer review only - http://bmjopen.bmj.com/site/about/guidelines.xhtml

plane block of isobaric bupivacaine with or without dexamethasone for bariatric patients undergoing laparoscopic vertical banded gastroplasty: a comparative study of different doses. *Ain-Shams Journal of Anaesthesiology* 2015;8(2):194-.

16. Albrecht E, Reynvoet M, Fournier N, et al. Dose-response relationship of perineural dexamethasone for interscalene brachial plexus block: a randomised, controlled, triple-blind trial. *Anaesthesia* 2019;74(8):1001-08. doi: 10.1111/anae.14650 [published Online First: 2019/04/12]

17. Bravo D, Aliste J, Layera S, et al. A multicenter, randomized comparison between 2, 5, and 8 mg of perineural dexamethasone for ultrasound-guided infraclavicular block. *Reg Anesth Pain Med* 2019;44(1):46-51. doi: 10.1136/rapm-2018-000032 [published Online First: 2019/01/15]

18. Liu J, Richman KA, Grodofsky SR, et al. Is there a dose response of dexamethasone as adjuvant for supraclavicular brachial plexus nerve block? A prospective randomized double-blinded clinical study. *J Clin Anesth* 2015;27(3):237-42. doi: 10.1016/j.jclinane.2014.12.004 [published Online First: 2015/02/02]

19. Turner JD, Henshaw DS, Weller RS, et al. Perineural dexamethasone successfully prolongs adductor canal block when assessed by objective pinprick sensory testing: A prospective, randomized, dose-dependent, placebo-controlled equivalency trial. *J Clin Anesth* 2018;48:51-57. doi: 10.1016/j.jclinane.2018.05.009 [published Online First: 2018/05/13]

20. Woo JH, Kim YJ, Kim DY, et al. Dose-dependency of dexamethasone on the analgesic effect of interscalene block for arthroscopic shoulder surgery using ropivacaine 0.5%: A randomised controlled trial. *Eur J Anaesthesiol* 2015;32(9):650-5. doi: 10.1097/eja.0000000000000213 [published Online First: 2015/01/21]

21. Kirkham KR, Jacot-Guillarmod A, Albrecht E. Optimal Dose of Perineural Dexamethasone to Prolong Analgesia After Brachial Plexus Blockade: A Systematic Review and Meta-analysis. *Anesth Analg* 2018;126(1):270-79. doi: 10.1213/ane.0000000000002488 [published Online First: 2017/09/19]

22. Albrecht E, Kern C, Kirkham KR. A systematic review and meta-analysis of

For peer review only - http://bmjopen.bmj.com/site/about/guidelines.xhtml

perineural dexamethasone for peripheral nerve blocks. *Anaesthesia* 2015;70(1):71-83. doi: 10.1111/anae.12823 [published Online First: 2014/08/16]

23. Bravo D, Aliste J, Layera S, et al. A multicenter, randomized comparison between 2, 5, and 8 mg of perineural dexamethasone for ultrasound-guided infraclavicular block. *Regional anesthesia and pain medicine* 2019;44(1):46-51. doi: 10.1136/rapm-2018-000032

24. Turner JD, Henshaw DS, Weller RS, et al. Perineural dexamethasone successfully prolongs adductor canal block when assessed by objective pinprick sensory testing: A prospective, randomized, dose-dependent, placebo-controlled equivalency trial. *Journal of Clinical Anesthesia* 2018;48((Turner J.D., jturner@wakehealth.edu; Henshaw D.S., dhenshaw@wakehealth.edu; Weller R.S., rweller@wakehealth.edu; Jaffe J.D., jjaffee@wakehealth.edu; Edwards C.J., chedward@wakehealth.edu; Reynolds J.W., jreynold@wakehealth.edu; Dobson S.W., sdobson@wakehealth.edu) Department of Anesthesiology, Wake Forest School of Medicine, Medical Center Boulevard, Winston-Salem, NC, United States):51-57. doi: 10.1016/j.jclinane.2018.05.009

25. Woo JH, Kim YJ, Kim DY, et al. Dose-dependency of dexamethasone on the analgesic effect of interscalene block for arthroscopic shoulder surgery using ropivacaine 0.5%: a randomised controlled trial. *European journal of anaesthesiology* 2015;32(9):650-55. doi: 10.1097/eja.0000000000000213

26. Hozo SP, Djulbegovic B, Hozo I. Estimating the mean and variance from the median, range, and the size of a sample. *BMC Med Res Methodol* 2005;5:13. doi: 10.1186/1471-2288-5-13 [published Online First: 2005/04/21]

27. Higgins J, Green SR. Cochrane Handbook for Systematic Review of InterventionsVersion 5.1.0. 2011

For peer review only - http://bmjopen.bmj.com/site/about/guidelines.xhtml

FIGURE TITLE

Figure 1. Template PRISMA flowchart

For peer review only

For peer review only - http://bmjopen.bmj.com/site/about/guidelines.xhtml

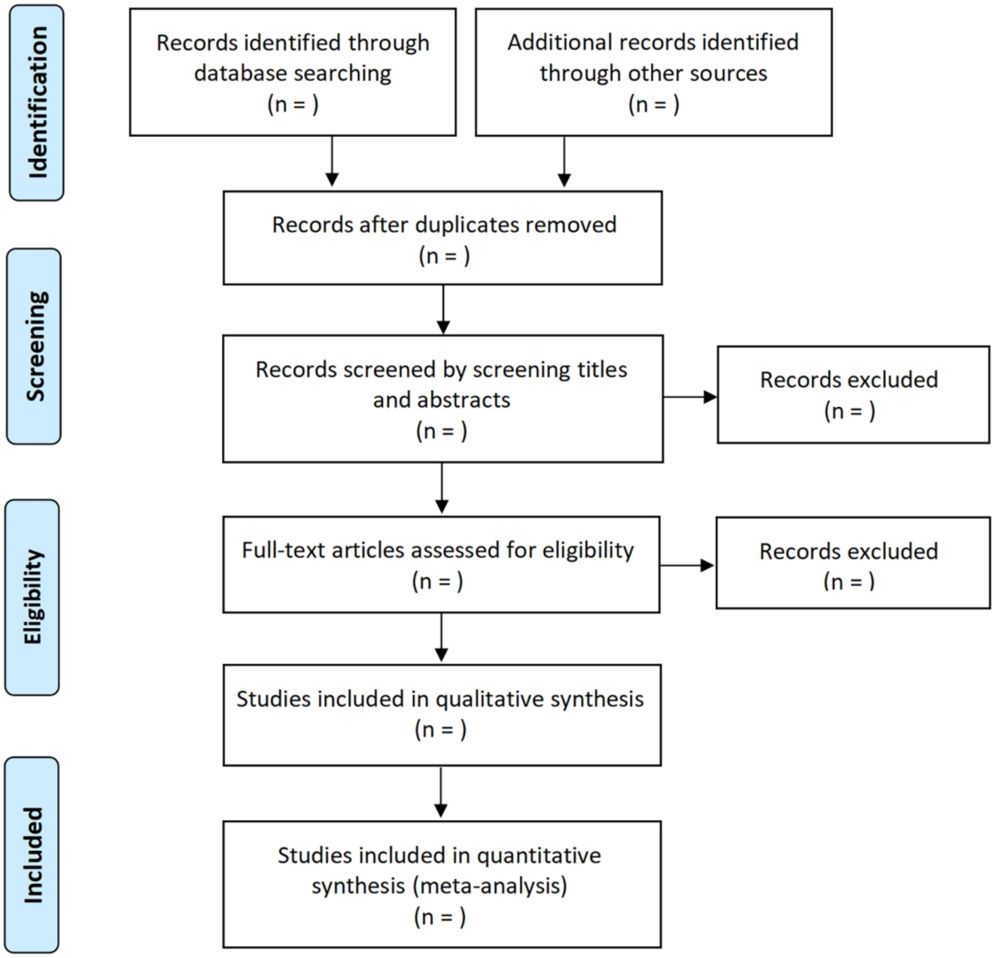

153x148mm (300 x 300 DPI)

Search strategies

EMBASE

#1 ‘Dexamethasone’/exp OR ‘Dexamethasone’:ti,ab,kw

#2 ‘Nerve block’/exp OR ‘Nerve block’:ti,ab,kw OR ‘Nerve blockade’:ti,ab,kw OR ‘Peripheral nerve blockade’:ti,ab,kw OR ‘Peripheral nerve block’:ti,ab,kw

#3 ‘Randomized controlled trial’/exp OR ‘Randomized controlled trial’:ti,ab,kw OR ‘Clinical study’:ti,ab,kw OR ‘Controlled clinical trial’:ti,ab,kw OR ‘Clinical trial’:ti,ab,kw OR ‘Randomized’:ti,ab,kw

#4 #1 AND #2 AND #3

Cochrane library Trials

#1 MeSH descriptor: [Dexamethasone| explode all trees

#2 (Dexamethasone):ti,ab,kw

#3 #1 OR#2

#4 (Nerve block):ti,ab,kw

#5 (Nerve blockade):ti,ab,kw

#6 (Peripheral nerve blockade):ti,ab,kw

#7 (Peripheral nerve block):ti,ab,kw

#8 #4 OR #5 OR #6 OR #7

#9 (Randomized controlled trial):ti,ab,kw

#10 (Clinical study):ti,ab,kw

#11 (Controlled clinical trial):ti,ab,kw

#12 (Clinical trial):ti,ab,kw

#13 (Randomized):ti,ab,kw

#14 #9 OR #10 OR #11 OR #12 OR #13

#15 #3 AND #8 AND #14

Web of science

#1 Dexamethasone (Topic)

#2 Nerve block (Topic) OR Nerve blockade (Topic) OR Peripheral nerve blockade

For peer review only

(Topic) OR Peripheral nerve block (Topic)

#3 Randomized controlled trial (Topic) OR Clinical study (Topic) OR Controlled clinical trial (Topic) OR Clinical trial (Topic) OR Randomized (Topic)

#4 #1 AND #2 AND #3

For peer review only

PRISMA-P (Preferred Reporting Items for Systematic review and Meta-Analysis Protocols) 2015 checklist: recommended items to address in a systematic review protocol*

Section and topic	Item No	Checklist item	Reported on page number
**ADMINISTRATIVE INFORMATION**			
Title:			
Identification	1a	Identify the report as a protocol of a systematic review	Page 1
Update	1b	If the protocol is for an update of a previous systematic review, identify as such	NA
Registration	2	If registered, provide the name of the registry (such as PROSPERO) and registration number	Page 2
Authors:			
Contact	3a	Provide name, institutional affiliation, e-mail address of all protocol authors; provide physical mailing address of corresponding author	Page 1
Contributions	3b	Describe contributions of protocol authors and identify the guarantor of the review	Page 9
Amendments	4	If the protocol represents an amendment of a previously completed or published protocol, identify as such and list changes; otherwise, state plan for documenting important protocol amendments	NA
Support:			
Sources	5a	Indicate sources of financial or other support for the review	Page 9
Sponsor	5b	Provide name for the review funder and/or sponsor	Page 9
Role of sponsor or funder	5c	Describe roles of funder(s), sponsor(s), and/or institution(s), if any, in developing the protocol	Page 9
**INTRODUCTION**			
Rationale	6	Describe the rationale for the review in the context of what is already known	Page 4
Objectives	7	Provide an explicit statement of the question(s) the review will address with reference to participants, interventions, comparators, and outcomes (PICO)	Page 4
**METHODS**			
Eligibility criteria	8	Specify the study characteristics (such as PICO, study design, setting, time frame) and report characteristics (such as years considered, language, publication status) to be used as criteria for eligibility for the review	Page 5
Information sources	9	Describe all intended information sources (such as electronic databases, contact with study authors, trial registers or other grey literature sources) with planned dates of coverage	Page 5
Search strategy	10	Present draft of search strategy to be used for at least one electronic database, including planned limits, such that it could be repeated	Page 5

For peer review only

Study records:			
Data management	11a	Describe the mechanism(s) that will be used to manage records and data throughout the review	Page 5
Selection process	11b	State the process that will be used for selecting studies (such as two independent reviewers) through each phase of the review (that is, screening, eligibility and inclusion in meta-analysis)	Page 5
Data collection process	11c	Describe planned method of extracting data from reports (such as piloting forms, done independently, in duplicate), any processes for obtaining and confirming data from investigators	Page 5-6
Data items	12	List and define all variables for which data will be sought (such as PICO items, funding sources), any pre-planned data assumptions and simplifications	Page 5
Outcomes and prioritization	13	List and define all outcomes for which data will be sought, including prioritization of main and additional outcomes, with rationale	Page 4
Risk of bias in individual studies	14	Describe anticipated methods for assessing risk of bias of individual studies, including whether this will be done at the outcome or study level, or both; state how this information will be used in data synthesis	Page 6
Data synthesis	15a	Describe criteria under which study data will be quantitatively synthesised	Page 6
	15b	If data are appropriate for quantitative synthesis, describe planned summary measures, methods of handling data and methods of combining data from studies, including any planned exploration of consistency (such as I^2, Kendall's τ)	Page 6
	15c	Describe any proposed additional analyses (such as sensitivity or subgroup analyses, meta-regression)	Page 6
	15d	If quantitative synthesis is not appropriate, describe the type of summary planned	Page 6
Meta-bias(es)	16	Specify any planned assessment of meta-bias(es) (such as publication bias across studies, selective reporting within studies)	Page 6
Confidence in cumulative evidence	17	Describe how the strength of the body of evidence will be assessed (such as GRADE)	Page 6

*** It is strongly recommended that this checklist be read in conjunction with the PRISMA-P Explanation and Elaboration (cite when available) for important clarification on the items. Amendments to a review protocol should be tracked and dated. The copyright for PRISMA-P (including checklist) is held by the PRISMA-P Group and is distributed under a Creative Commons Attribution Licence 4.0.**

From: Shamseer L, Moher D, Clarke M, Ghersi D, Liberati A, Petticrew M, Shekelle P, Stewart L, PRISMA-P Group. Preferred reporting items for systematic review and meta-analysis protocols (PRISMA-P) 2015: elaboration and explanation. BMJ. 2015 Jan 2;349(jan02 1):g7647.

For peer review only
